# Exploring the Fit Between the Outputs of Freely Available Medication Adherence Apps and Users’ Needs: Mixed Methods Study

**DOI:** 10.2196/68919

**Published:** 2025-12-16

**Authors:** Kirstin Messner, Vanessa Sutter, Samuel Allemann, Isabelle Arnet

**Affiliations:** 1 Department Pharmaceutical Sciences University of Basel Basel Switzerland; 2 TopPharm Hirschen Apotheke Magden Magden Switzerland

**Keywords:** desirable features, implementation, medication adherence, mHealth, needs assessment, outputs, users’ perspective

## Abstract

**Background:**

Medication nonadherence is a significant barrier to therapy success. Smartphone apps represent reasonable tools for simple adherence-enhancing interventions. Many adherence apps are available in app stores with diverse content, quality, and outputs. We define “output of an adherence app” as the processing and visualization of data recorded by the user and related to adherence. In 2016, Santo et al defined 5 desirable features in the output of adherence apps: tracking history, charts, statistics, rewards, and an exportable file. With this, a reference point to evaluate outputs of adherence apps was delivered. Identifying and fulfilling users’ needs are essential when developing an adherence app for patients’ self-management and professional adherence services, such as therapy support provided by health care professionals (HCPs).

**Objective:**

We aimed to investigate the smartphone app market regarding desirable features in the outputs, explore the users’ needs, and evaluate the concordance.

**Methods:**

We searched for smartphone adherence apps in the 2 largest commercial app repositories by using keywords. Search results were screened for eligibility by applying inclusion and exclusion criteria. Eligible, freely available apps were tested regarding desirable features in their output. We conducted 2 focus groups and a cross-sectional online survey to explore users’ needs. Survey participants rated their desire for features on a 7-point Likert scale. Focus groups were analyzed using the previously reported framework method. Descriptive statistics were calculated by median and IQR or mean and SD. We compared survey subgroups with a 2-tailed *t* test. A *P* value <.05 was considered statistically significant.

**Results:**

We screened 80 apps for eligibility and included 9 in our analyses. All desirable features were present, with tracking history being the most frequent feature (in 8/9 apps). Other desirable features were observed in 3 or fewer of the apps. Eight individuals participated in the 2 focus groups. During the focus groups, a total of 13 categories of desired features emerged. All 5 desirable features were rated as important in adherence apps. Three additional features were mentioned: (1) professional feedback regarding therapy or intake course, (2) additional recommendations based on intake course, and (3) option to discuss the data with an HCP. A total of 42 individuals participated in the online survey. Tracking history was the most desired (mean rating of 5.29) and rewards the least desired feature (mean rating of 2.81) in the output. There was ambivalence regarding professional feedback, statistics, and charts. Participants with or without regular medication use showed no significant differences.

**Conclusions:**

The outputs delivered by freely available smartphone adherence apps only partly match users’ needs. Users showed a special interest in the interpretation of their data with an HCP. Therefore, adherence apps cannot substitute for the HCP but can be used to enhance current patient care.

## Introduction

Medication nonadherence is known to be a significant barrier to therapy success, leading to higher mortality and morbidity rates as well as increased health care costs [1]. According to the World Health Organization (WHO) in 2003, adherence to medication for chronic diseases averages around 50% [1]. Approximately half of medication nonadherence is unintentional, caused by forgetfulness, unawareness of incorrect intake behavior, or an overly complex treatment regimen, among others [2,3].

In the era of digitalization and mobile health (mHealth) where the smartphone is ubiquitous, smartphone apps offer new possibilities for simple interventions on adherence difficulties [4]. In 2023 in Switzerland, over 96% of the population were in possession of a smartphone [5], with increasing tendencies in the next years. Therefore, apps constitute a reasonable tool for interventions to address medication adherence difficulties up to optimizing adherence.

Currently, many medication adherence apps are available in commercial app stores with diverse content, functionalities, and quality [6]. Most of them are intended for the sustainable improvement of medication self-management. Currently, it is unclear whether they can achieve this objective [7]. Various systematic reviews regarding adherence apps report a significant improvement in medication adherence with their use [8-10]. Since forgetfulness is one major barrier to adherence [3], reminding patients is a continuously pursued method in the management of adherence. This is done by adherence apps with the so-called reminder function, which often consists of a sound and a push message sent from the app to the mobile phone of the patient at scheduled intake times.

In 2018, Ahmed et al [11] analyzed medication adherence apps regarding their used adherence strategies and grouped them into 3 categories: reminder, educational, and behavioral strategies. While a reminder was the most commonly used strategy (92% of all tested apps), behavioral (45%) and educational strategies (9%) were underrepresented. Bailey et al [6] also reported an integrated reminder function in about 90% of the available health apps. A meta-analysis of 16 studies on chronic diseases reported an increase in adherence from 50% to 67.8% with the use of a reminder function, demonstrating its optimizing effect on medication adherence [12].

Overall, while the effects of the reminder function are extensively investigated, little is known about the usefulness and benefits of the educational and behavioral strategies in adherence apps. Educational strategies in adherence apps aim to increase patients’ knowledge about their medication and can include features such as feedback or professional recommendations based on adherence data [13]. Behavioral strategies seek to induce behavioral change through features such as tracking or reward systems [13]. Among all available features, we selected those that involve the processing, visualization, and interpretation of adherence data recorded by users in the app to define the “output of an adherence app.” In 2016, Santo et al [14] published a stepwise process to identify high-quality medication adherence apps. Based on prior research and literature, they defined 20 so-called “desirable features” in adherence apps and categorized them into practical and functionality features. Among the 17 functionality features, a total of 5 concerned the output and were: tracking history, charts, statistics, rewards and an exportable file. Santo et al [14] delivered a reference point that can be used to investigate further the features in the output of adherence apps and to draw conclusions on the quality of adherence apps’ outputs.

Moreover, in times where implementation sciences become more important [15] and the involvement of patients and public is required in any development [16], identifying the needs of the target group and matching the intervention to the end users are key aspects for the successful implementation of any intervention [17,18]. Therefore, awareness and knowledge of the users’ needs are essential for the development of an adherence app that may be used for patients’ self-management as well as for professional services, such as a pharmacist-led adherence service.

In summary, adherence apps’ outputs as well as the users’ perspective and needs have been insufficiently investigated so far. With this study, we aimed to investigate the current adherence app market regarding 5 desirable features in the output reported by Santo et al [14], explore users’ needs, and evaluate the concordance. Our overarching research question is: to what extent do freely available medication adherence apps deliver outputs that match users’ needs.

## Methods

### App Search Strategy and Selection Process

An app search was conducted in October 2023 in the Swiss Apple App Store and Google Play Store by using the following key words: *medication adherence, medication reminder, medication tracker,* and *medication management*. The key words were selected based on previous research regarding systematic medication adherence app searches [14] and adapted to our context and interest. Predefined inclusion and exclusion criteria were applied to the search results. Inclusion criteria were: in English or German language, freely available or free version available, compatible with iOS and Android, last update latest in 2022, ready to use after opening (no bugs or other technical problems), targets the human population, minimum 4-stars rating in the store, and minimum 10 users’ ratings in the store. Exclusion criteria were: contains only a general alarm (eg, alarm clock), limited to one specific medication (eg, birth control pill), limited to one specific health condition (eg, asthma), limited to one specific population (eg, women), focuses on lifestyle or fitness, includes only a game, delivers only medical information, or is provided by a health care professional (HCP) for their own patients.

We followed a pragmatic approach by evaluating the highest ranked and therefore most visible adherence apps in the app stores and screened the first 20 app results per search term for eligibility. Eligibility screening was performed by KM based on the information available in the app description and screenshots in the stores. The reporting of the app search was informed by the PRISMA (Preferred Reporting Items for Systematic reviews and Meta-Analyses) statement, where applicable (Multimedia Appendix 1) [19].

### App Analysis Regarding Desirable Features

Apps that matched the inclusion criteria were analyzed by KM regarding the features in their output. If a free version was available with the option for a paid or professional version, only the cost-free features were included in the analysis. Eligible apps were downloaded, and timepoints for the intakes of an illustrative medication were scheduled, that is, ibuprofen 400 mg, 2 times daily, for 3 consecutive days. The reminder function was enabled if available in the app. In a random order, half of the scheduled medication intakes were confirmed, and the remaining intakes were intentionally skipped to mimic missed doses. After 3 days, the provided adherence outputs were evaluated by listing all observed desirable features per app in a Microsoft Excel spreadsheet. We calculated the frequency of occurrence of the 5 desirable features in the output defined by Santo et al [14]. Additionally, we assessed the following characteristics of included apps based on their app store description and websites: rating, number of ratings, registration as a medical device, and involvement of HCP in the development process.

### Focus Groups

Since literature suggests that 2 to 3 focus groups are likely to capture at least 80% of themes on a topic [20], we conducted 2 focus groups to explore users’ perspective and needs regarding the output of adherence apps. Purposive sampling was used within the acquaintance of the researchers. Participants were contacted by phone, messenger, and email. Requirements for participation were owning a smartphone and having experience with mHealth apps. Participation was voluntary. Each group included the moderator (KM), 2 note-takers (VS and IA), and 4 participants. No prior relationship was established between KM and the participants. A total of 5 participants were recruited from the personal network of VS and 1 participant from the personal network of IA. A semistructured guide was developed and approved by KM, VS, and IA. The guide consisted of a first part where participants were invited to talk about their experiences with mHealth and adherence apps, and a second part where an example of a freely available adherence app with all 5 desirable features was presented. In each focus group, the researchers introduced themselves (name, current position, research focus), presented the focus group process, and outlined the research purpose. To characterize the participants, we assessed age, sex, job, and nationality.

All discussions were audio recorded. Participants were asked to give their opinion on the different features and to rate which of the presented features was useful or important to them. For the rating, participants lifted a green card to indicate agreement and a red card to indicate disagreement. Voting scores were announced orally and written down by the note-takers. After every rating, participants were invited to explain the reasons for their decision. The focus groups were reported in accordance with the COREQ (Consolidated Criteria for Reporting Qualitative Research) checklist (Multimedia Appendix 2) [21].

### Online Survey

We conducted a cross-sectional online survey. A 3-item survey was developed in German language that inquired about (1) the participants’ characteristics, (2) one main question “Which of the following features would you desire in an app to manage your medication intake?” and (3) a free comment section. A list of the 5 desirable features from literature, expanded by the findings from the focus groups with illustrative examples and explanations, was provided. Participants rated their desire for each feature on a 7-point Likert-Scale from 1 (absolutely not desirable) to 7 (absolutely desirable); all items were mandatory.

We used the secure web application REDCap (Research Electronic Data Capture; Vanderbilt University) for the design of the online survey and the data collection. The online survey was pretested and piloted with 39 pharmacy students in their first year of the master’s program, regarding technical functionality and linguistic clarity. Mean age of the master’s students was 24 years. A total of 32 (82%) students were female. Pretesting participants were smartphone owners, and 11 participants (28%) took regular medication. Differences from the pretest participants to the survey sample population include a younger age, a higher proportion of females, and a closer connection to the health care sector.

We disseminated the survey link by email or mobile messenger services to approximately 60 eligible participants in October 2023 through snowball sampling. Purposive sampling was used within the acquaintance of the researchers (KM, VS, IA). Access to the survey was restricted to individuals who were directly provided with the survey link. The link was valid for 4 weeks. To reach a heterogeneous group of potential adherence app users, the only eligibility criterion for participation was the possession of a smartphone. The participants of the focus groups were excluded from participation in the online survey since they provided the feature suggestions and were biased. The online survey was reported in accordance with the Checklist for Reporting Results of Internet E-Surveys (CHERRIES; Multimedia Appendix 3) [22].

### Data Analysis

The focus groups were analyzed according to the framework method by Gale et al [23]. All audio files were transcribed verbatim by VS and coded by MV. The codes referred to desired features of an adherence app that were mentioned by participants. The codes were grouped in categories according to the underlying adherence-optimizing strategy that is reminder, behavioral, or educational, if applicable. For the analysis of the online survey, only completed responses were considered. As multiple submissions could not be technically prevented, we checked the dataset for potential duplicates in participant characteristics. Duplicates were not considered for the analysis.

Descriptive statistics were calculated by frequencies (in %), mean and SD, or median and IQR. When averaging the points of the 7-point Likert-Scale rating, a value in the range of ±5% of the neutral middle position of 4, that is, between 3.80 and 4.20, was defined as “ambivalent.” We used 2-tailed *t* tests to compare subgroups (participants with vs without regular medication) in the online survey. All calculations were performed with Microsoft Excel. A *P* value of <.05 was considered statistically significant.

### Ethical Considerations

This study did not require ethics approval according to Swiss law and the University of Basel’s policy. We ensured that all aspects of the study were conducted in compliance with applicable legal and institutional guidelines. Written informed consent was obtained from all participants in the focus groups and online survey. Focus group participants were compensated with CHF 25 (US $31) for participation. The survey was conducted anonymously and did not allow any identification of participants.

## Results

### App Search and Feature Analysis

#### Characteristics of the Included Apps

A total of 80 apps were screened for eligibility. Nine apps out of the 80 (11%) apps met the inclusion and exclusion criteria. Five out of the 9 (56%) apps had a paid version besides the freely available version. The median star rating was 4.5 (IQR 4.0-4.6). The number of ratings in the stores ranged from 12 to 230,450.

Of these 9 apps, a registration as a medical device was mentioned for 2 (22%) apps in the store description or on the app’s webpage. The involvement of an HCP in the app’s development was indicated for 4 (44%) apps (2 with physicians, 2 with pharmacists) and of a pharmaceutical company for 1 (11%) app. The remaining 4 (44%) apps provided no information regarding their development processes.

#### Frequency of the Desirable Features in the Output

All 5 desirable features according to Santo et al [14] were observed with a median of 1 feature per app (IQR 1-2). The tracking history was observed in 8 out of 9 apps and was the most frequent (89%). All other desirable features were observed in 3 or fewer of the tested apps (Table 1). Charts were the least observed feature with only 1 app out of 9 (11%).

**Table 1:** Number and proportion of apps with corresponding features (N=9). Number and percentage of the 5 desirable features according to Santo et al [14] observed in the outputs of 9 adherence smartphone apps.

Feature	Frequency, n (%)
Tracking history	8 (89)
Statistics	3 (33)
Rewards	2 (22)
Exportable file	2 (22)
Charts	1 (11)

### Focus Groups

A total of 8 individuals accepted to take part in the focus groups that were conducted in person in 2023 at the University of Basel on March 20 (4 participants) and March 21 (4 participants). All participating individuals had previous experience with mHealth apps. The mean age was 25.3 (SD 3.5) years in the first focus group and 41.8 (SD 20.3) years in the second focus group. A total of 2 (50%) participants in the first focus group and 3 (75%) participants in the second focus group were female. A total of 2 (25%) participants in the second group were working in health care. The nationality of all 4 (100%) participants in the first focus group was German. In the second focus group 2 participants were German and 2 participants were Swiss (50% each). The focus groups lasted 80 and 100 minutes, respectively.

The app functionalities that were desired by the participants were classified into 13 categories, of which 6 (46%) addressed outputs of adherence apps and 7 (54%) concerned general functions (Table 2). Four out of the 13 (31%) categories were assigned to behavioral strategies, 3 (23%) to educational strategies, and 1 (8%) category to reminder strategy.

**Table 2:** Categories of the desired features of a medication adherence app mentioned by participants of 2 focus group discussions, grouped into features in the output (n=6) and general functions (n=7).

Section and category of desired features		Adherence-optimizing strategy
**Features in the output**		
	1. Additional recommendations or information based on intake course	Educational
	2^a^. Exportable file (eg, to the physician)	N/A^b^
	3. Option to discuss the adherence data with a health care professional	Educational
	4^a^. Overview on the intakes and therapy course in form of statistics, history, or charts	Behavioral
	5. Professional feedback regarding therapy or intake course	Educational
	6^a^. Rewards (eg, collection of points or streaks)	Behavioral
**General functions**		
	7^a^. Ad-free	N/A^b^
	8^a^. Discretion (eg, no medication name in push messages), data security, and anonymity	N/A^b^
	9. Management of several medications including an interaction check	N/A^b^
	10. Motivational inputs (gamification, messages)	Behavioral
	11^a^. Reminder function (eg, push notifications)	Reminder
	12. Scan function for medication, therapy plans, or prescriptions	N/A^b^
	13. Symptom tracking and well-being tracking	Behavioral

^a^Concordance with all features mentioned by Santo et al [14].

^b^Not applicable.

All 5 desirable features were mentioned by the participants and rated as important to exist in a medication adherence app. Three new features in the output were mentioned, which were: (1) professional feedback regarding therapy or intake course, (2) additional recommendations or information based on intake course, and (3) option to discuss the adherence data with an HCP (Table 2). One participant stated regarding professional feedback on therapy course:

I would appreciate it if feedbacks were provided. For example, if I experience side effects, I would receive a response like: ‘Yes, that's normal and included in 90% of the reported side effects, you don't need to worry.’ or ‘Please go to the pharmacy.’ or ‘Please contact your doctor.’

Participant 3, male, 27 years old

Another participant advocated for additional recommendations and reminders.

It would be helpful to get a reminder or some additional educational material. Something like a simple overview of the ‘dos and don’ts’ with the most important information about what could influence the therapy, both positively and negatively.

Participant 4, female, 29 years old

Following the voting, 75% of the participants in both groups agreed on the usefulness of the additional 3 features, especially in case of a worsening therapy course.

These findings led to a final list of 8 desired features selected by adherence app users, which are: tracking history, charts, statistics, rewards, exportable file, professional feedback in the app, additional recommendations and information, and option to discuss the data with an HCP. They were used for the online survey.

### Online Survey

#### Characteristics of the Participants

A total of 50 out of the 60 invited participants took part in the survey, corresponding to a participation rate of 83%. Eight surveys were excluded from the analysis due to incomplete answers (n=7; completion rate of 86%) and conspicuous responses due to a general aversion against adherence apps (n=1), as indicated in the section titled “Free Comment Section.” A total of 42 surveys were analyzed. Participants showed a median time for completion of 3.3 (IQR 2.3-5.4) minutes.

The mean age of the participants was 53 (SD 19.1, range 25-86) years and 22 of the 42 (52%) participants were female. Regular medication was taken by 26 (62%) participants, with 14 (33%) participants taking 1-2 medications, 8 (19%) participants taking 3-4 medications, and 4 (10%) participants taking 5 or more medications. No participant was currently using an app for their medication management.

#### Desired Features

Overall, the participants rated the tracking history as the most desired feature in the output of an adherence app and rewards as the least desired feature. Ambivalence was observed for the features professional feedback, statistics, and charts (Table 3). There were no significant differences in perception between participants with and without regular medication intakes (mean rating of participants with regular medication vs without regular medication: tracking history 5.62 vs 4.75; exportable file 5.08 vs 4.44; option to discuss with HCP 4.50 vs 4.19; additional Recommendations 4.27 vs 4.13; professional feedback 4.19 vs 3.94; statistics 3.92 vs 4.19; charts 3.65 vs 4.44; and rewards 2.35 vs 3.56).

Half of the participants rated the option to discuss the data with an HCP and additional recommendations with 5 points or more, indicating a strong desire for those features (Table 4).

**Table 3:** Participant ratings of desire for 8 app features (n=42), presented as mean (SD) on a 7-point Likert scale, where 1 represents “no desire” and 7 represents “absolute desire.”

Feature	Mean (SD)
Tracking history	5.29 (1.09)
Exportable file	4.83 (1.72)
Option to discuss with an HCP^a^	4.38 (1.79)
Additional recommendations	4.21 (1.75)
Professional feedback	4.10 (1.75)
Statistics	4.02 (1.81)
Charts	3.95 (1.86)
Rewards	2.81 (1.63)

^a^HCP: health care professional.

**Table 4:** Scores of 42 survey participants regarding the 8 features in the output of adherence apps, presented as number and frequency—n (%)—for each Likert-scale point from 1 (absolutely not desirable) to 7 (absolutely desirable).

Feature	Likert-scale point, n (%)						
	1 (absolutely not desirable)	2 (not desirable)	3 (somewhat not desirable)	4 (neutral)	5 (somewhat desirable)	6 (desirable)	7 (absolutely desirable)
Tracking history	1 (2.4)	2 (4.8)	6 (14.3)	3 (7.1)	6 (14.3)	11 (26.2)	13 (31)^a^
Exportable file	3 (7.1)	1 (2.4)	7 (16.7)	1 (2.4)	14 (33.3)^a^	9 (21.4)	7 (16.7)
Option to discuss with an HCP^b^	4 (9.5)	3 (7.1)	5 (11.9)	9 (21.4)^a^	8 (19)	8 (19)	5 (11.9)
Additional recommendations	4 (9.5)	4 (9.5)	6 (14.3)	6 (14.3)	13 (31)^a^	5 (11.9)	4 (9.5)
Professional feedback	4 (9.5)	5 (11.9)	7 (16.7)	6 (14.3)	9 (21.4)^a^	9 (21.4)^a^	2 (4.8)
Statistics	3 (7.1)	8 (19)	5 (11.9)	10 (23.8)^a^	6 (14.3)	5 (11.9)	5 (11.9)
Charts	5 (11.9)	5 (11.9)	7 (16.7)	10 (23.8)^a^	5 (11.9)	5 (11.9)	5 (11.9)
Rewards	11 (26.2)^a^	11 (26.2)	7 (16.7)	4 (9.5)	6 (14.3)	3 (7.1)	0 (0)

^a^Indicates the highest frequency.

^b^HCP: health care professional.

#### Free Comment Section

A total of 10 out of the 42 (24%) survey participants suggested 1 or more additional features in the free comment section that they would desire in a medication adherence app, which were: a reminder function (4, 10% participants), a direct contact or data export to their physician (2, 5% participants each), suggestions for alternative medications in case of unavailability of their usual medication (1, 2% participants), and the possibility to reorder medication if needed (1, 2% participants). Comments on the app structure concerned local data storage (2, 5% participants) and that the app should be kept simple (“keep it simple and stupid”; 2, 5% participants).

## Discussion

### Principal Results

Our results regarding the features in the output of 9 medication adherence apps indicate that the majority of freely available medication adherence apps only partly deliver the desirable outputs. The delivery of a tracking history was the strategy most often pursued by app developers to present adherence data to the user. This observation seems to coincide with the users’ needs, as our online survey indicated the tracking history to be the most desired and most important feature for potential users. In a similar but opposite way, the features less present in adherence apps were adherence data processing or visualization in the form of charts, statistics or rewards, and were also the features least needed by survey participants. On the contrary, regarding sharing data, for example, through an exportable file or a direct exchange with HCP, only 2 apps out of 9 included such a feature in their cost-free version, although our participants quoted the availability of such additional features as highly important.

Our findings align with the findings from Santo et al [14] where tracking history was by far the most frequent output feature in the tested adherence apps, while the other 4 features were only present in one fourth or fewer of them. This observation leads us to the conclusion that in the last few years—despite a rapidly growing and evolving field [24]—the outputs of adherence apps remained predominantly unchanged. Nevertheless, the 9 adherence apps we tested ranked the highest in the app stores, although the desired output features were lacking. This situation may have several reasons.

First, from the users’ perspective, one reason may be a lack of understanding regarding the importance of good adherence behavior. Delivering statistics such as a percentage of taken doses or charts may not be tangible for the common user and therefore not of deep interest in an adherence app. This hypothesis is strengthened by our survey results, which revealed a need by half of the participants for professional inputs or feedback based on their adherence data or treatment course, and especially for the possibility to share their data with an HCP either by an exportable file or a direct discussion with an HCP. In 2014, Hilliard et al [25] described a similar result when asking users for their preferences and design recommendation for an mHealth app to promote cystic fibrosis self-management. Participants were especially interested in communicating with their HCP between clinic visits and improving care by giving HCPs access to their data through the app. In summary, users seem to be more interested in the clinical meaning of their adherence behavior and impact on their treatment course rather than getting feedback on their performance with medication use, for example, in the form of statistics. This conclusion provides a rationale for the usefulness and importance of professional adherence services that, for example, could use mHealth apps as a practical and easily accessible solution to collect adherence data.

Second, users might be more interested in other app functions than adherence data outputs, for example, in the reminder function. This consideration is supported by the findings from Park et al [26], where users especially appreciated the feature of helpful reminders and notifications. The desire for a reminder function was also expressed and rated as a key feature in our focus groups, as well as by several participants in the online survey in the free comment section. Such a reminder function was integrated in all of the 9 tested apps, which aligns with the findings from prior app content analyses [6,11] and indicates the awareness of the current mHealth market about this strong users’ desire.

Third, although our analysis was limited to the non–disease-specific apps, noteworthy differences were observed in the literature for disease-specific apps. A systematic review of freely available mobile apps for breast cancer survivorship and self-management found that apps specific for this condition primarily focus on app features such as educational material, symptom tracking, and social networking [27]. In contrast, adherence-enhancing features such as reminders or tracking were underrepresented in those apps. This suggests that app developers may prioritize different features depending on the primary target audience. Universal, non–disease-specific apps tend to emphasize simple general app features such as reminders, tracking, or the ability to export data, whereas disease-specific apps place greater emphasis on condition-related features. Since ease of use is a key factor valued by users of non–disease-specific adherence apps [28], maintaining a lean and simple app structure might be an intentional decision by app developers.

We did not assess the association between app ranking and frequency of key features. This ranking is—besides keyword matching—primarily determined by factors such as active user engagement, number of downloads, retention rates, and ratings [29]. Findings from a previous study indicate that higher-rated apps (≥4 stars) included desirable features such as tracking history and data export/sharing features more often than lower-rated apps (<4 stars) [30]. Similar trends were observed for incentives, complex medication instructions, and medical social networking [30]. A similar study found that consumer ratings were most strongly correlated with general features, such as update frequency, charges, and absence of advertisements [31]. Among these, the strongest individual factor associated with ratings was whether the app was free of charge. Therefore, it remains debatable whether the desirable features have a significant impact on user ratings, while the cost-free nature of the apps included in the analysis and their reminder function appear to be the most influential factors contributing to high ratings.

However, besides reminder and general app functions, the mHealth market should not underestimate the usefulness of other adherence strategies such as educational and behavioral strategies, as the majority of desired features identified in the focus groups were related to these strategies. Those strategies in adherence apps could further enhance the adherence of their users, since it is reported that knowledge of the medication and the disease itself is one contributing factor to higher adherence rates [32,33]. In 2013, a systematic review and meta-analysis showed that feedback on recent dosing history can increase adherence in patients [34]. In practice, this could be done by professional feedback from the app or, again, feedback from an HCP—as suggested by our focus group participants and desired by half of our survey participants. Delivering such feedback features could further exploit the potential of adherence apps to improve adherence on multiple levels in the future. Moreover, by integrating more desired features, adherence apps could constitute a useful and appropriate tool for patients’ medication self-management or for an HCP-driven adherence support. In addition to purely improving adherence, user-friendlier apps can lead to overall higher satisfaction and willingness to use such an app, particularly in the long term for chronic diseases. A systematic review on factors influencing adherence to mHealth apps in noncommunicable diseases highlighted that ongoing contact or telecoaching with HCPs, as well as personalized feedback, positively affected app adherence [35]. Therefore, with user-friendlier apps, the likelihood of achieving a sustainable implementation of adherence apps increases, especially in a professional setting such as an adherence service delivered by an HCP. Further research in the real-world setting is necessary to gain deeper knowledge about the implementability of an app-based professional adherence service.

### Strengths and Limitations

This project had several strengths. First, the demographics of our participants were highly heterogeneous, such as the age distribution, especially in the online survey. Therefore, our participants were more likely to represent the general population. Nevertheless, the generalizability of our findings is limited due to the small sample size. To obtain representative results, a larger, population-based survey might be necessary. Second, the majority of participants in the online survey took regular medication, which matches the main target group of adherence apps. Their perception of desirable features in an mHealth app is therefore of special interest and relevance to this project. Third, during the development process, the online survey was tested and piloted with pharmacy students in the first year of master’s program. In this way, we could ensure flawless technical functionality and general comprehensibility of the survey.

We acknowledge some limitations. First, the presented results of the app test are limited in their generalizability to the whole range of available adherence apps, since we included only freely available apps. Thus, the presence of desirable features in the apps’ whole market may be underestimated. Missing desirable features such as the option to contact an HCP or an exportable file are often present in the paid or pro version of an app, especially in those apps that are designed with HCPs or are registered as a medical device. However, patients may prefer a freely available app for mHealth to support them in their medication use, especially when they are newly taking medicines. Thus, our results are valuable. Second, we excluded apps designed for specific diseases from the analysis. As noted in the literature, disease-specific apps such as for diabetes management or cancer care often include a tailored combination of features that specifically address the needs of patients with the respective condition [27,36,37]. Our results may therefore be limited in their ability to reflect the needs of patients with specific conditions. Although general features of adherence apps may be helpful to promote user engagement, future research could focus on stratifying adherence feature requirements based on disease type and management complexity to ensure optimal support and effectiveness. Third, our analysis is restricted to the Swiss app stores. We cannot rule out that results might be different for other regions. Fourth, no participants in the survey had experience using an adherence app, which implies that there was no practical knowledge on adherence apps and the usefulness of different features. Therefore, the perception of our participants may differ from that of actual users.

### Conclusions

Freely available adherence apps only partly deliver adherence data outputs that match users’ needs. Therefore, it is conceivable that apps currently do not fully exploit their potential in optimizing adherence and user engagement. Our participants showed a special interest in the interpretation of their entered adherence data, for example, in form of a discussion with their HCP and recommendations from the app, which are features primarily available exclusively in the paid and pro versions of the apps. Adherence apps, either as free or paid version, are not able and neither should be intended to replace HCPs; instead, they could be used to complement current patient care by integrating apps into the treatment setting. More research is needed to further investigate the integration of adherence apps into medical and pharmaceutical workflow.

In the future, and for an effective and sustainable implementation of adherence apps in patients’ self-management or professional adherence services, further research is required regarding the feasibility and acceptance of adherence apps in the real-world setting, from the patients’ and HCPs’ perspectives. Besides proving the effectiveness of reminder functions, other adherence strategies should be brought more into focus, such as adherence data outputs or educational inputs, and their effects on patients’ adherence and user engagement.

## Appendix

Multimedia Appendix 1 PRISMA checklist.

Multimedia Appendix 2 COREQ checklist.

Multimedia Appendix 3 Checklist for Reporting Results of Internet E-Surveys (CHERRIES).

## Abbreviations

CHERRIES: Checklist for Reporting Results of Internet E-Surveys
COREQ: Consolidated Criteria for Reporting Qualitative Research
HCP: health care professional
mHealth: mobile health
PRISMA: Preferred Reporting Items for Systematic reviews and Meta-Analyses
REDCap: Research Electronic Data Capture
WHO: World Health Organization

## Data Availability

All datasets generated and analyzed during this study are not publicly available due to data protection and privacy regulations but may be obtained from the corresponding author upon reasonable request.
