# Supplementary material for: Exploring the Fit Between the Outputs of Freely Available Medication Adherence Apps and Users’ Needs: Mixed Methods Study
Source: JMIR Mhealth Uhealth. 2025 Dec 16;13:e68919. doi: 10.2196/68919 (PMC12754584; doi:10.2196/68919)
Supplement: Multimedia Appendix 1 [file mhealth_v13i1e68919_app1.docx]

| **Section and Topic** | **Item #** | **Checklist item** | **Location where item is reported** |
| --- | --- | --- | --- |
| **TITLE** | | |  |
| Title | 1 | Identify the report as a systematic review. | Due to the mixed method approach, the app review is not stated in the titel |
| **ABSTRACT** | | |  |
| Abstract | 2 | See the PRISMA 2020 for Abstracts checklist. | Due to the mixed-methods approach, the abstract includes the most relevant aspects of the app search and review. |
| **INTRODUCTION** | | |  |
| Rationale | 3 | Describe the rationale for the review in the context of existing knowledge. | The rationale is outlined in the background section, explicitly in the fourth and fifth paragraphs. |
| Objectives | 4 | Provide an explicit statement of the objective(s) or question(s) the review addresses. | Statement is provided in the last paragraph of the background. |
| **METHODS** | | |  |
| Eligibility criteria | 5 | Specify the inclusion and exclusion criteria for the review and how studies were grouped for the syntheses. | Inclusion and exclusion criteria are specified in the chapter "App search strategy and selection process". |
| Information sources | 6 | Specify all databases, registers, websites, organisations, reference lists and other sources searched or consulted to identify studies. Specify the date when each source was last searched or consulted. | Searched app stores and dates are specified in the chapter "App search strategy and selection process". |
| Search strategy | 7 | Present the full search strategies for all databases, registers and websites, including any filters and limits used. | All used key words are presented in the chapter "App search strategy and selection process". |
| Selection process | 8 | Specify the methods used to decide whether a study met the inclusion criteria of the review, including how many reviewers screened each record and each report retrieved, whether they worked independently, and if applicable, details of automation tools used in the process. | Selection process is described in the second paragraph of the chapter "App search strategy and selection process". |
| Data collection process | 9 | Specify the methods used to collect data from reports, including how many reviewers collected data from each report, whether they worked independently, any processes for obtaining or confirming data from study investigators, and if applicable, details of automation tools used in the process. | Methods for extraction of the available features is specified in the chapter "App analysis regarding desirable features" |
| Data items | 10a | List and define all outcomes for which data were sought. Specify whether all results that were compatible with each outcome domain in each study were sought (e.g. for all measures, time points, analyses), and if not, the methods used to decide which results to collect. | We assessed the presence of the five desirable app features. See chapter "App analysis regarding desirable features". |
|  | 10b | List and define all other variables for which data were sought (e.g. participant and intervention characteristics, funding sources). Describe any assumptions made about any missing or unclear information. | We assessed app characteristics such as star rating, number of ratings, registration as medical device, and involvement of HCP in the development process. See chapter "App analysis regarding desirable features". |
| Study risk of bias assessment | 11 | Specify the methods used to assess risk of bias in the included studies, including details of the tool(s) used, how many reviewers assessed each study and whether they worked independently, and if applicable, details of automation tools used in the process. | Not applicable. |
| Effect measures | 12 | Specify for each outcome the effect measure(s) (e.g. risk ratio, mean difference) used in the synthesis or presentation of results. | All undertaken measures are specified in the chapter "Data analysis" |
| Synthesis methods | 13a | Describe the processes used to decide which studies were eligible for each synthesis (e.g. tabulating the study intervention characteristics and comparing against the planned groups for each synthesis (item #5)). | Not applicable. |
|  | 13b | Describe any methods required to prepare the data for presentation or synthesis, such as handling of missing summary statistics, or data conversions. | Not applicable. |
|  | 13c | Describe any methods used to tabulate or visually display results of individual studies and syntheses. | Methods to tabulate the results per app are described in the chapter "App analysis regarding desirable features". |
|  | 13d | Describe any methods used to synthesize results and provide a rationale for the choice(s). If meta-analysis was performed, describe the model(s), method(s) to identify the presence and extent of statistical heterogeneity, and software package(s) used. | Not applicable. |
|  | 13e | Describe any methods used to explore possible causes of heterogeneity among study results (e.g. subgroup analysis, meta-regression). | Not applicable. |
|  | 13f | Describe any sensitivity analyses conducted to assess robustness of the synthesized results. | Not applicable. |
| Reporting bias assessment | 14 | Describe any methods used to assess risk of bias due to missing results in a synthesis (arising from reporting biases). | Not applicable. |
| Certainty assessment | 15 | Describe any methods used to assess certainty (or confidence) in the body of evidence for an outcome. | Not applicable. |
| **RESULTS** | | |  |
| Study selection | 16a | Describe the results of the search and selection process, from the number of records identified in the search to the number of studies included in the review, ideally using a flow diagram. | Described in the results section "App search and feature analysis" within the chapter "Characteristics of the included apps". |
|  | 16b | Cite studies that might appear to meet the inclusion criteria, but which were excluded, and explain why they were excluded. | Not applicable. |
| Study characteristics | 17 | Cite each included study and present its characteristics. | Overall characteristics of the apps is reported in the chapter "Characteristics of the included apps". |
| Risk of bias in studies | 18 | Present assessments of risk of bias for each included study. | Not applicable. |
| Results of individual studies | 19 | For all outcomes, present, for each study: (a) summary statistics for each group (where appropriate) and (b) an effect estimate and its precision (e.g. confidence/credible interval), ideally using structured tables or plots. | For each desirable feature, the frequency is presented in the chapter "Frequency of the desirable features in the output" |
| Results of syntheses | 20a | For each synthesis, briefly summarise the characteristics and risk of bias among contributing studies. | Not applicable. |
|  | 20b | Present results of all statistical syntheses conducted. If meta-analysis was done, present for each the summary estimate and its precision (e.g. confidence/credible interval) and measures of statistical heterogeneity. If comparing groups, describe the direction of the effect. | No additional statistical analysis was performed. |
|  | 20c | Present results of all investigations of possible causes of heterogeneity among study results. | Not applicable. |
|  | 20d | Present results of all sensitivity analyses conducted to assess the robustness of the synthesized results. | Not applicable. |
| Reporting biases | 21 | Present assessments of risk of bias due to missing results (arising from reporting biases) for each synthesis assessed. | Not applicable. |
| Certainty of evidence | 22 | Present assessments of certainty (or confidence) in the body of evidence for each outcome assessed. | Not applicable. |
| **DISCUSSION** | | |  |
| Discussion | 23a | Provide a general interpretation of the results in the context of other evidence. | Interpretation of the results is provided in the chapter "Principle results", explicitly in the second, third and forth paragraphs. |
|  | 23b | Discuss any limitations of the evidence included in the review. | Limitations are discussed in the second paragraph of the chapter "Strengths and Limitations" |
|  | 23c | Discuss any limitations of the review processes used. | Limitations are discussed in the second paragraph of the chapter "Strengths and Limitations" |
|  | 23d | Discuss implications of the results for practice, policy, and future research. | Practical implications for future research and app development are discussed in the last paragraph of the chapter "Principle results" |
| **OTHER INFORMATION** | | |  |
| Registration and protocol | 24a | Provide registration information for the review, including register name and registration number, or state that the review was not registered. | Not applicable. |
|  | 24b | Indicate where the review protocol can be accessed, or state that a protocol was not prepared. | Not applicable. |
|  | 24c | Describe and explain any amendments to information provided at registration or in the protocol. | Not applicable. |
| Support | 25 | Describe sources of financial or non-financial support for the review, and the role of the funders or sponsors in the review. | No funding was received for the conduct of this study. Non-financial support is described in the Acknowledgments. |
| Competing interests | 26 | Declare any competing interests of review authors. | All authors have no conflicts of interests to declare. See chapter "Conflicts of interest" |
| Availability of data, code and other materials | 27 | Report which of the following are publicly available and where they can be found: template data collection forms; data extracted from included studies; data used for all analyses; analytic code; any other materials used in the review. | Reported in the Data availability statement. |
