# Supplementary material for: Exploring the Fit Between the Outputs of Freely Available Medication Adherence Apps and Users’ Needs: Mixed Methods Study
Source: JMIR Mhealth Uhealth. 2025 Dec 16;13:e68919. doi: 10.2196/68919 (PMC12754584; doi:10.2196/68919)
Supplement: Multimedia Appendix 2 [file mhealth_v13i1e68919_app2.docx]

| **Domain 1: Research team and reflexivity** | | |
| --- | --- | --- |
| **Personal characteristics** | | |
| 1. Interviewer/facilitator | Which author(s) conducted the interview or focus group? | KM carried out both focus groups as moderator. VS and IA assissted as note taker in both groups. |
| 2. Credentials | What were the researcher’s credentials? (e.g. PhD, MD) | KM has a MSc and VS obtained her MSc six months after the focus groups. IA has a PhD and PD. |
| 3. Occupation | What was their occupation at the time of the study? | KM was a PhD student, IA a senior researcher and VS a master student. |
| 4. Gender | Was the researcher male or female? | KM, VS and IA were female. |
| 5. Experience and training | What experience or training did the researcher have? | KM and IA had previous experience in this methodology. |
| **Relationship with participants** | | |
| 6. Relationship established | Was a relationship established prior to study commencement? | No prior relationship was established between KM and participants. Five participants were recruited from the personal network of VS and one from IA. |
| 7. Participant knowledge of the interviewer | What did the participants know about the researcher? (e.g. personal goals, reasons for doing the research) | Participants knew where the researchers worked and the reasons for doing the research. They were aware of the incorporation of the results into VS’s master’s thesis. |
| 8. Interviewer characteristics | What characteristics were reported about the interviewer/facilitator? (e.g. bias, assumptions, reasons and interests in the research topic) | The three researchers had an interest in the research. The research focus of KM's PhD was on medication adherence apps and research focus of IA on adherence in general. |
| **Domain 2: Study design** | | |
| **Theoretical framework** | | |
| 9. Methodological orientation and theory | What methodological orientation was stated to underpin the study? (e.g. grounded theory, discourse analysis, ethnography, phenomenology, content analysis) | Qualitative content analysis: Focus group analysis was based on the Framework Method by Gale et al. |
| **Participant selection** | | |
| 10. Sampling | How were participants selected? (e.g. purposive, convenience, consecutive, snowball) | Purposive sampling was used within the acquaintance of the researchers. Requirements for participation were owning a Smartphone and having experience with mHealth apps. The researchers contacted the participants directly. |
| 11. Method of approach | How were participants approached? (e.g. face to face, telephone, mail, e-mail) | Participants were contacted via personal contact (phone, messenger, e-mail) |
| 12. Sample size | How many participants were in the study? | 8 participants took part in two focus groups, 4 participants each. |
| 13. Non-participation | How many people refused to participate or dropped out? Reasons? | Numbers of refusals were not recorded. |
| **Setting** | | |
| 14. Setting of data collection | Where was the data collected? (e.g. home, clinic, workplace) | Focus groups took place at the Department of Pharmaceutical Sciences of the University of Basel. |
| 15. Presence of non-participants | Was anyone else present besides the participants and researchers? | No |
| 16. Description of sample | What are the important characteristics of the sample? (e.g. demographic data, date) | We assessed age, sex, job and nationality. |
| **Data collection** | | |
| 17. Interview guide | Were questions, prompts, guides provided by the authors? Was it pilot tested? | A focus group guide with questions was developed and used during the sessions. The guide guide reviewed and approved by all three researchers. |
| 18. Repeat interviews | Were repeat interviews carried out? If yes, how many? | No |
| 19. Audio/visual recording | Did the research use audio or visual recording to collect the data? | Focus groups were audio-recorded and written notes were taken. |
| 20. Field notes | Were field notes made during and/or after the interview or focus group? | Written notes were taken of the voting results. |
| 21. Duration | What was the duration of the interviews or focus group? | Focus group duration ranged from 80 to 100 min |
| 22. Data saturation | Was data saturation discussed? | Data saturation was not discussed |
| 23. Transcripts returned | Were transcripts returned to participants for comment and/or correction? | No |
| **Domain 3: Analysis and findings** | | |
| **Data analysis** | | |
| 24. Number of data coders | How many data coders coded the data? | MV coded the data |
| 25. Description of the coding tree | Did authors provide a description of the coding tree? | Final categories are shown in Table 1. |
| 26. Derivation of themes | Were themes identified in advance or derived from the data? | Themes were derived from the data and referred to desired features |
| 27. Software | What software, if applicable, was used to manage the data? | Microsoft Excel |
| 28. Participant checking | Did participants provide feedback on the findings? | No |
| **Reporting** | | |
| 29. Quotations presented | Were participant quotations presented to illustrate the themes/findings? Was each quotation identified? (e.g. participant number) | Participant quotations with participant number were presented |
| 30. Data and findings consistent | Was there consistency between the data presented and the findings? | We aimed to present the study findings clearly and consistently to accurately reflect the data collected. Since the categories were derived from explicit statements about desired features, we assert robustness of the results. |
| 31. Clarity of major themes | Were major themes clearly presented in the findings? | Major themes are clearly presented in Table 1. |
| 32. Clarity of minor themes | Is there a description of diverse cases or discussion of minor themes? | Coding and category building was clear without any discussions regarding minor themes |
