# Supplementary material for: Exploring the Fit Between the Outputs of Freely Available Medication Adherence Apps and Users’ Needs: Mixed Methods Study
Source: JMIR Mhealth Uhealth. 2025 Dec 16;13:e68919. doi: 10.2196/68919 (PMC12754584; doi:10.2196/68919)
Supplement: Multimedia Appendix 3 [file mhealth_v13i1e68919_app3.docx]

| **Item Category** | **Checklist Item** | **Explanation** |  |
| --- | --- | --- | --- |
| **Design** | Describe survey design | Describe target population, sample frame. Is the sample a convenience sample? (In “open” surveys this is most likely.) | Cross-sectional online survey. The target population were potential users of adherence apps, therefore every individual owning a smartphone could participate in the survey. Purposive sampling was used. |
| **IRB (Institutional Review Board) approval and informed consent process** | IRB approval | Mention whether the study has been approved by an IRB. | Our study did not require ethics approval according to Swiss law and the University of Basel’s policy. We have ensured that all aspects of the study were conducted in compliance with applicable legal and institutional guidelines. |
|  | Informed consent | Describe the informed consent process. Where were the participants told the length of time of the survey, which data were stored and where and for how long, who the investigator was, and the purpose of the study? | Informed consent was obtained from all participants prior to the start of the online survey. Information about the estimated completion time (maximum 10 minutes), the purpose of the study, and the investigators’ contact details was provided in the invitation email and the survey introduction. |
|  | Data protection | If any personal information was collected or stored, describe what mechanisms were used to protect unauthorized access. | The survey was conducted anonymously, no personal information that allows any identification of participants was collected |
| **Development and pre-testing** | Development and testing | State how the survey was developed, including whether the usability and technical functionality of the electronic questionnaire had been tested before fielding the questionnaire. | We developed a 3-item survey in German language with the main question “Which of the following features would you desire in an app to manage your medication intake?”. We provided the list of the five desirable features from literature, expanded by the findings from the focus groups. Participants rated their desire for each feature on a 7-point Likert-Scale from 1 (absolutely not desirable) to 7 (absolutely desirable). The online survey was pretested with 39 pharmacy students in their first Master year regarding technical functionality and linguistic clarity. |
| **Recruitment process and description of the sample having access to the questionnaire** | Open survey versus closed survey | An “open survey” is a survey open for each visitor of a site, while a closed survey is only open to a sample which the investigator knows (password-protected survey). | Access to the survey was restricted to individuals who were directly provided with the survey link, the link was not made publicly available. |
|  | Contact mode | Indicate whether or not the initial contact with the potential participants was made on the Internet. (Investigators may also send out questionnaires by mail and allow for Web-based data entry.) | We disseminated the survey link via e-mail and mobile messenger services |
|  | Advertising the survey | How/where was the survey announced or advertised? Some examples are offline media (newspapers), or online (mailing lists – If yes, which ones?) or banner ads (Where were these banner ads posted and what did they look like?). It is important to know the wording of the announcement as it will heavily influence who chooses to participate. Ideally the survey announcement should be published as an appendix. | A purposive sampling strategy was employed limited to individuals within the acquaintance of the researchers.. No external recruitment efforts, such as public announcements or advertisements, were undertaken. |
| **Survey administration** | Web/E-mail | State the type of e-survey (eg, one posted on a Web site, or one sent out through e-mail). If it is an e-mail survey, were the responses entered manually into a database, or was there an automatic method for capturing responses? | Data collection was conducted using the secure web application REDCap. Participants entered their responses directly into the platform, where all data were securely stored. |
|  | Context | Describe the Web site (for mailing list/newsgroup) in which the survey was posted. What is the Web site about, who is visiting it, what are visitors normally looking for? Discuss to what degree the content of the Web site could pre-select the sample or influence the results. For example, a survey about vaccination on a anti-immunization Web site will have different results from a Web survey conducted on a government Web site | The survey was not posted on a website. |
|  | Mandatory/voluntary | Was it a mandatory survey to be filled in by every visitor who wanted to enter the Web site, or was it a voluntary survey? | The survey was voluntary. |
|  | Incentives | Were any incentives offered (eg, monetary, prizes, or non-monetary incentives such as an offer to provide the survey results)? | No incentives were offered for participation in the survey. |
|  | Time/Date | In what timeframe were the data collected? | The survey link was valid for 4 weeks. |
|  | Randomization of items or questionnaires | To prevent biases items can be randomized or alternated. | No items were randomized or alternated. |
|  | Adaptive questioning | Use adaptive questioning (certain items, or only conditionally displayed based on responses to other items) to reduce number and complexity of the questions. | No adaptive questioning was used. |
|  | Number of Items | What was the number of questionnaire items per page? The number of items is an important factor for the completion rate. | The survey consisted of 3-items: i) the participants’ characteristics (6 questions); ii) a matrix with the main question “Which of the following features would you desire in an app to manage your medication intake?” (8 features for rating) and iii) a free comment section. |
|  | Number of screens (pages) | Over how many pages was the questionnaire distributed? The number of items is an important factor for the completion rate. | The questionnaire was distributed over 3 pages. One page for each item. |
|  | Completeness check | It is technically possible to do consistency or completeness checks before the questionnaire is submitted. Was this done, and if “yes”, how (usually JAVAScript)? An alternative is to check for completeness after the questionnaire has been submitted (and highlight mandatory items). If this has been done, it should be reported. All items should provide a non-response option such as “not applicable” or “rather not say”, and selection of one response option should be enforced. | All items were mandatory. Users could not proceed to the next page without completing all required fields. There was no non-response option. |
|  | Review step | State whether respondents were able to review and change their answers (eg, through a Back button or a Review step which displays a summary of the responses and asks the respondents if they are correct). | Respondents were able to revise their answers using a back button prior to submission. Once the survey was submitted, no further changes could be made. |
| **Response rates** | Unique site visitor | If you provide view rates or participation rates, you need to define how you determined a unique visitor. There are different techniques available, based on IP addresses or cookies or both. | We did not determine unique visitors. Participation rate was calculated based on participating individuals compared to invited individuals. |
|  | View rate (Ratio of unique survey visitors/unique site visitors) | Requires counting unique visitors to the first page of the survey, divided by the number of unique site visitors (not page views!). It is not unusual to have view rates of less than 0.1 % if the survey is voluntary. | The number of individuals who viewed the survey page was not tracked; therefore, no view rate could be calculated. |
|  | Participation rate (Ratio of unique visitors who agreed to participate/unique first survey page visitors) | Count the unique number of people who filled in the first survey page (or agreed to participate, for example by checking a checkbox), divided by visitors who visit the first page of the survey (or the informed consents page, if present). This can also be called “recruitment” rate. | We did not determine unique visitors. Participation rate was calculated based on participating individuals compared to invited individuals. |
|  | Completion rate (Ratio of users who finished the survey/users who agreed to participate) | The number of people submitting the last questionnaire page, divided by the number of people who agreed to participate (or submitted the first survey page). This is only relevant if there is a separate “informed consent” page or if the survey goes over several pages. This is a measure for attrition. Note that “completion” can involve leaving questionnaire items blank. This is not a measure for how completely questionnaires were filled in. (If you need a measure for this, use the word “completeness rate”.) | Completion rate was calculated based on participants submitting the questionnaire compared to participants giving informed consent compared to |
| **Preventing multiple entries from the same individual** | Cookies used | Indicate whether cookies were used to assign a unique user identifier to each client computer. If so, mention the page on which the cookie was set and read, and how long the cookie was valid. Were duplicate entries avoided by preventing users access to the survey twice; or were duplicate database entries having the same user ID eliminated before analysis? In the latter case, which entries were kept for analysis (eg, the first entry or the most recent)? | Not applicable. |
|  | IP check | Indicate whether the IP address of the client computer was used to identify potential duplicate entries from the same user. If so, mention the period of time for which no two entries from the same IP address were allowed (eg, 24 hours). Were duplicate entries avoided by preventing users with the same IP address access to the survey twice; or were duplicate database entries having the same IP address within a given period of time eliminated before analysis? If the latter, which entries were kept for analysis (eg, the first entry or the most recent)? | Not applicable. |
|  |  |  |  |
|  |  |  |  |
|  |  |  |  |
|  |  |  |  |
|  |  |  |  |
|  | Log file analysis | Indicate whether other techniques to analyze the log file for identification of multiple entries were used. If so, please describe. | Not applicable. |
|  | Registration | In “closed” (non-open) surveys, users need to login first and it is easier to prevent duplicate entries from the same user. Describe how this was done. For example, was the survey never displayed a second time once the user had filled it in, or was the username stored together with the survey results and later eliminated? If the latter, which entries were kept for analysis (eg, the first entry or the most recent)? | From the technical side, the possibility of multiple entries could not be ruled out. We checked dataset for potential duplicates in the participant characteristics. |
| **Analysis** | Handling of incomplete questionnaires | Were only completed questionnaires analyzed? Were questionnaires which terminated early (where, for example, users did not go through all questionnaire pages) also analyzed? | Only completed online surveys were considered for the analysis. |
|  | Questionnaires submitted with an atypical timestamp | Some investigators may measure the time people needed to fill in a questionnaire and exclude questionnaires that were submitted too soon. Specify the timeframe that was used as a cut-off point, and describe how this point was determined. | We did not measure the time for completion. |
|  | Statistical correction | Indicate whether any methods such as weighting of items or propensity scores have been used to adjust for the non-representative sample; if so, please describe the methods. | Not applicable. |
